# Regionally-structured explanations behind area-level populism: An update to recent ecological analyses

**DOI:** 10.1371/journal.pone.0229974

**Published:** 2020-03-12

**Authors:** Roger Beecham, Nick Williams, Alexis Comber

**Affiliations:** 1 School of Geography, University of Leeds, Leeds, United Kingdom; 2 Business School, University of Leeds, Leeds, United Kingdom; Augusta University, UNITED STATES

## Abstract

Heavy geographic patterning to the 2016 Brexit vote in UK and Trump vote in US has resulted in numerous ecological analyses of variations in area-level voting behaviours. We extend this work by employing modelling approaches that permit regionally-specific associations between outcome and explanatory variables. We do so by generating a large number of regional models using penalised regression for variable selection and coefficient evaluation. The results reinforce those already published in that we find associations in support of a ‘left-behind’ reading. Multivariate models are dominated by a single variable—levels of *degree-education*. Net of this effect, ‘secondary’ variables help explain the vote, but do so differently for different regions. For Brexit, variables relating to material disadvantage, and to a lesser extent structural-economic circumstances, are more important for regions with a strong industrial history than for regions that do not share such a history. For Trump, increased material disadvantage reduces the vote both in global models and models built mostly for Southern states, thereby undermining the ‘left-behind’ reading. The reverse is nevertheless true for many other states, particularly those in New England and the Mid-Atlantic, where comparatively high levels of disadvantage assist the Trump vote and where model outputs are more consistent with the UK, especially so for regions with closer economic histories. This pattern of associations is exposed via our regional modelling approach, application of penalised regression and use of carefully designed visualization to reason over 100+ model outputs located within their spatial context. Our analysis, documented in an accompanying github repository, is in response to recent calls in empirical Social and Political Science for fuller exploration of subnational contexts that are often controlled out of analyses, for use of modelling techniques more robust to replication and for greater transparency in research design and methodology.

## Introduction

Recent support for right-wing populist agendas—the Brexit vote in the United Kingdom (UK), the election of Donald Trump in the United States (US)—has invited much academic and popular discussion (e.g. [1]). Theoretical juxtaposition of these two events is not straightforward. Parallels between race [2] and nationalism [3] require careful discussion given the diverging cultural histories between UK and US and ‘populism’ is itself an overused and conceptually stretched term [4]. Brexit may represent a new socio-economic cleavage that is reshaping British politics [9], but it is also informed by decades of British euroscepticism; equally, electoral support for Trump may be symptomatic of a new populism, but cannot be easily separated from Republicanism and its various guises, which also informed the vote [5].

There has been more consolidated discussion around place-based factors and voting behaviour, supported by an observed geographic patterning to the two electoral outcomes, which in both cases appears systematic [4]. Here, the focus is on structural changes associated loosely with globalisation and the resulting concentration of economic and cultural activity in particular regions and elsewhere, a story of decline—depressed wages, limited opportunities and immobile populations [6]. Recent ecological analyses provide empirical support to this ‘left-behind’ places reading, with models presented that explain area-level variation in both the Brexit and Trump votes through variables describing employment structure, education levels and migration profiles [4], or variables closely related to these themes [7].

In several of these studies, area-level variation is explained using a range of selected variables and comparatively little analysis attention is paid to the structure of model residuals: that is, net of area-level demographics and other context, *where* unexplained variation is concentrated and why the pattern of concentration might exist. This latter omission is important as a country as large as US, with its population of 325 million spread over 3,142 counties and 51 states, is inevitably diverse. It is likely that two US counties that belong to the same US state are more similar in socio-economic and voting terms than two randomly sampled counties from entirely different states. Global models that do not take this dependency into account may misrepresent or hide associations between variables. In extreme cases, the associations between voting and socio-economic characteristics—their relative importance and even direction of effect—may change between global and local levels of analysis. Different combinations of variables may, then, be more relevant to explaining variation in voting behaviour in particular regions than others. This has been explored in studies of the Brexit vote and that regionally-specific explanations were demonstrated to exist [8] provides further justification for regional comparison between the US and GB cases: it is conceivable that certain parts of the US and GB exhibit similar patterns of association and therefore similar categories of area-level explanation between place-based factors and voting variation.

By extending recent analyses of populist votes with techniques that allow for regional context, this study explores regionally-specific categories of explanation behind the Trump and Brexit votes. Results data for 2016 EU referendum in UK and Presidential Election in US are collected along with data that describe area-level circumstances, informed by the ‘left-behind’ [4, 9] interpretations of these votes. Multilevel models are first employed to quantify the extent and nature of regional structuring of associations between area-level demographics and the Brexit and Trump votes. Regionally-specific explanations, with discrete combinations of variables and effects, are then explored by generating a large number of regional models—for 48 states of mainland US and District of Columbia and 11 regions of Great Britain.

Generating models for each state of mainland US and region of GB creates a challenge both for selecting variables—we wish to allow for the fact that certain variables may be more relevant to particular regions than others—and model comparison. We therefore use penalised regression [10], a class of regression techniques typically applied to ‘wide’ datasets incorporating many variables but increasingly used in social science domains on more traditional datasets [11], for variable regularisation and selection. The different categories of regional explanation, differing sets of explanatory variables with differing priorities suggested, are then characterised through carefully designed visualization.

As well as empirical contributions, this work has implications for Social and Political Science methodology. Our analysis, further documented via a github repository, responds to recent calls for greater introspection into regional and subnational contexts and trends in statistical models [12], for application of nascent regression modelling techniques more robust to replication and that more fully represent uncertainty information [11] and for greater transparency in research design and methodology [11–13].

## Ecological analyses of 2016’s populist votes

### Global ecological models

Both in public and scholarly discussion, recent populist right-wing sentiment has been associated with places ‘left-behind’ by globalisation. A broad set of ecological analyses has provided empirical support to these claims, exploring the extent to which variation in area-level socio-economic outcomes is associated with area-level populist vote shares. For UK’s 2016 EU referendum vote, Goodwin & Heath [9] and Becker et al. [14] compared results data against variables describing the age, ethnicity, migration structure and economic outcomes of residents and found education profiles to have the largest effect on area-level differences in the Leave vote. Lesser effects were observed for variables describing the age and migration structure of areas [9], historical employment structures (dependence on manufacturing) and incomes [14, 15].

That such studies are based on data that are publicly available and unproblematic (aggregated count data) allows independent analyses to be compared and findings corroborated, but also for comparison to be made across political events. Essletzbichler et al. [4] analysed results data from the 2016 EU Referendum in the UK (the Brexit vote), the Presidential Elections in the US (the Trump vote) and Austria (the populist right-wing Hofer vote) along with data describing the demographic composition and structural-economic circumstances of administrative areas. They found some consistency across the three countries: areas with an industrial history, with comparatively low migration and with high unemployment tend to exhibit high populist vote shares. Obschonka et al. [7] combined results and area-level composition data with a slightly different dataset—a large-scale survey aggregated to area-level from which the Big Five personality traits [16] can be inferred. Positive associations between area-level Brexit and Trump vote shares and area-level *neurotic* personality traits were observed and an association in the opposite direction for personality traits related to *openness*.

### Exploring, quantifying and adjusting for local and regional context

Most often, ecological regression models such as those above exhibit residuals with spatial dependency. For certain parts of a country, a model will overestimate an outcome given the relationship implied by global associations between explanatory and outcome variables; for other parts, the outcome will be underestimated. This has been shown for ecological analyses that model area-level variation in the Brexit vote (e.g. [8]). One explanation for spatial dependency in model residuals is the presence of subnational processes that constrain a variable’s range. Area-level income, for example, is likely to be bounded to economic regions and failing to adjust for this may result in imprecise approximations of associations between explanatory and outcome variables. A further explanation is that associations between variables—the underlying processes—might vary for different parts of the country. For example, high levels of international migration might inform political attitudes differently in different parts of a country; areas that are economically and socially successful might regard migration more positively than those for which high-value jobs and services are in short supply. Global models that fail to allow for this possibility may misrepresent the processes they are trying to capture and hide more subtle insights into phenomena.

Often the range problem is adjusted for by modelling some subnational (and regional) level as a fixed effect. In their analysis of area-level variation in the Brexit vote, Goodwin & Heath [9] created dummy variables for Scotland and London and Essletzbichler et al. [4] similarly created dummy variables for the seven US divisions in their analysis of the Trump vote. Coefficients are estimated to quantify the unexplained variation in each regional dummy and the effect of the explanatory variables are estimated outside of this. Such approaches offer a means of adjusting for, or ‘control[ing] away’ [12, 17], the unexplained effect of regional variation. They allow generalisable claims to be made around explanatory variables since parameter estimates are not complicated by the more messy regional heterogeneity.

Multilevel modelling provides an alternative framework for incorporating regional controls. A hierarchical structure is identified—for an analysis of the Brexit vote (e.g. [8, 18]) that 380 Local Authority Districts (LADs) in Great Britain at level 1 sit within 11 Government Office Regions (GOR) at level 2—and the standard regression model extended to recognise this two-level structure. For *random intercept* multilevel models, the effect of explanatory variables on the outcome is assumed to be constant (the *slope* is fixed), but the model intercept is allowed to vary (is *random*) on region (GOR in this case). Multilevel models can be further extended such that *both* the intercept and slope are allowed to vary. The assumption here is that not only may the distribution and range of variables be regionally constrained, but that the effect of explanatory variables on the outcome—the association between variables—are subject to change. This is a more unique addition in that it accepts that regionally-distinct effects may exist and provides a framework for exploring and quantifying those effects.

### Extending analyses into local and regional context

The extent to which ecological studies attend to sub-national scale effects may depend on disciplinary focuses [12]. For some, the regional effect, whether modelled as a random intercept or fixed effect dummy variable, might be regarded as a necessary ‘nuisance’ term incorporated in order to estimate coefficients that generalise across contexts (e.g. the effect of socio-economics on the outcome net of the regional effects). For others, the structure and organisation of regional intercepts or dummy regression coefficients might be worthy of investigation in and of itself. In studying the Brexit vote and its aftermath, Harris & Charlton [18], Beecham et al. [8] and Johnston et al. [19] variously describe regional and more local structure in model residuals. In Beecham et al. [8] the possibility of regionally diverging associations between variables is addressed explicitly. Through a multilevel modelling framework, and also local modelling approaches, Beecham et al. [8] found that not only did the size of associations between area-level socio-economic variables and the Brexit vote diverge between regions, but that in some cases their direction shifted between positive and negative associations.

This work extends the analysis presented in Beecham et al. [8] through a more explicit and detailed examination of regional structure via penalised regression and, importantly, introducing a comparison with the 2016 Trump vote—an event occurring in a country much larger and more diverse than UK. Whilst ecological analyses have already been published comparing these political events [4, 7], none have yet incorporated modelling techniques that support detailed exploration of regionally varying effects. In collecting an equivalent set of explanatory variables covering US and UK, consistency in area-level explanations is anticipated in line with Obschonka et al. [7] and Essletzbichler et al. [4]. Deploying modelling techniques that estimate different effects between variables for different parts of the US and UK, we nevertheless expect to observe some regionally-specific explanation—and characterising this variation and its geography will be an important analysis task. Our assumption is that for parts of the US and GB with similar social-economic histories, and particularly former industrial regions ‘left-behind’ by globalisation, there will be similar categories of explanation (similar combinations of effect contributed by our explanatory variables). The scale at which we explore regionally-varying effects is UK Government Office Region (GOR) and US state—the 380 Local Authority Districts of GB are grouped into 11 GORs and the 3,108 counties of mainland US are grouped into 48 states and District of Columbia. It should be noted that no strong theoretical basis is used for selecting these regional units; these are simply a function of the administrative hierarchies at which data are recorded. Certainly compared with states of US, GB GORs are reasonably artificial boundaries. A key focus of our analysis is therefore in comparison of different model outputs: that we use a principled approach to variable selection (penalised regression) and are careful when characterising model outputs to analyse global and regional outputs concurrently is a key aspect to our analysis.

## Data and analysis design

### Voting data and scale of analysis

For the US, results from 2012 and 2016 presidential elections have been published at county-level by The Guardian and townhall.com respectively and collated by Tony McGovern. Data are available only at state-level for Alaska. For the UK, the Electoral Commission has published results data for 2016 EU referendum by Local Authority District (LAD); data are available only at Government Office Region-level (GOR) for Northern Ireland. Our analysis is therefore based on counties in mainland US (Alaska and Hawaii are excluded) and LADs in Great Britain (Northern Ireland is excluded). This amounts to 3,108 US counties spread across 48 States (and District of Columbia) and 380 GB LADs spread across 11 GORs.

We assume equivalence between US counties and states and GB LADs and GORs respectively. With a median population size according to 2010 Census of 26,011, US counties are generally smaller in population terms than LADs (median population size of 143,358 according to 2011 Census). However, whilst both administrative delineations are insensitive to variation in population density, this is especially so for US counties. According to the 2010 Census the largest county is Los Angeles with a population of 9,818,605, the smallest is Loving County, Texas with a population size of 82. The corresponding figures for GB LADs are 2,146,090 for Birmingham, West Midlands and 4,406 for Isles of Scilly, South West (2011 Census). This has implications for our analysis; we smooth over much variation in densely populated US counties and underrepresent processes there, whilst the less densely populated counties are overrepresented.

### Outcome variables

For 2016 EU Referendum dataset, the number of votes for Leave is calculated as a proportion of all Leave and Remain votes cast by LAD. When exploring geographic patterns in voting preference we express this quantity (*net-Leave*) as a signed margin in favour of Leave|Remain. For 2016 US Presidential election the two-party proportional vote share by US county (*net-Trump*) is calculated. Again, this is expressed as a signed margin in favour of Trump|Clinton. We also calculate a second outcome variable for 2016 Trump vote (as do [7]): the net change in two-party vote share between the 2012 and 2016 elections. This might be regarded loosely as part of the vote that is due not merely to Trump being a Republican candidate. Comparison of model outputs on these three outcome variables may be instructive for making inferences around area-level populism and ‘left-behind’ places. Since Brexit did not divide neatly into historical British political party affiliations [20], we speculate that there may be greater equivalence between the *shift-Trump* variable and *net-Leave* than with *net-Trump*, which is more heavily conflated with the Republican vote.

### Explanatory variables

Similar to the existing literature, we collect candidate ‘explanatory’ variables *a priori* based on the narrative of ‘left-behind’ places. Particularly for regions once monopolised by single-industries, a scarcity of secure employment, outmigration of more mobile populations and stagnant or falling health and wealth outcomes has created a sense of decline that is geographically distinctive [6]. The essence of the ‘left behind’ reading is that this geography is reflected in recent populist sentiment, with the additional observation of a widening between satellite towns and core cosmopolitan cities [21].

The variables selected on this interpretation are summarised in Table 1. Full variable definitions are provided here, but to aid interpretation we also provide shortened variable names, in *itallics*, and organise variables according to the underlying concepts they are assumed to represent. All variables are expressed as a share of the total population in each US county or GB LAD, with the exception of median *household income* and *population density*.

**Table 1:** Explanatory variables selected based on existing analysis and ostensibly the ‘left-behind’ interpretation, also selected to support comparison between US and GB contexts. Variables are expressed as a proportion of the total population in each US county or GB LAD, with the exception of *household income* and *population density*.

concept	variable name	definition	source US | GB
structural-economic	*leisure & hospitality* *manufacturing loss* ^1^ *transport, trade & utilities*	% in leisure & hospitality jobs net % change in manufacturing jobs % in transport, trade & utilities jobs	2010 | 2011 Census 1970-2010 | 1971-2011 Census 2010 | 2011 Census
socio-demographic	*degree-educated* *household income* *not good health* | *poverty*	% with Bachelors level or higher median household income % not in good health | living in poverty	2010 | 2011 Census 2016 ACS^2^ | 2015 GDHI^3^ 2010 | 2011 Census
age-migrant mix	*foreign born* *older adults*	% not UK-born | not US-born % age 65+ years	2010 | 2011 Census 2010 | 2011 Census
metropolitan / satellite	*population density*	log of population density	2010 | 2011 Census

^1^ 1970 US Census data published via [25]; 1971 GB Census data harmonised to 2011 LADs using approach published in [26].

^2^ American Community Survey

^3^ Gross Disposible Household Income

Changes in the relative share of employment accounted for by manufacturing since 1970s (*manufacturing loss*) and the current share in employment sectors recently identified as vulnerable to automation (*transport, trade & utilities*—[22]) were used to quantify the extent to which areas are structurally exposed to globalisation. The share of employment in *leisure & hospitality* was selected as a variable that represents post-industrial employment in a subtle way; unlike employment in ‘knowledge-intensive’ activities (e.g. *business services*) it is not heavily correlated with socio-demographics and may capture locales that have transitioned from traditional industries but into employment that is lower value and potentially precarious. In terms of socio-demographics, *degree-educated* was selected since highly-educated workers epitomise the post-industrial ‘knowledge economy’ [23] and whilst *household income* is reasonably correlated with this, following an analysis of individual-level survey data post the Brexit vote [24], we expect a more complicated pattern of association with income and populist voting after controlling for levels of education. The share of local populations identifying with *not good health* (GB) and living in *poverty* (US) were selected as proxies of material disadvantage. Assuming equivalence between these two variables is somewhat problematic in that the 2010 US Census measure—the proportion in a county judged to be living in *poverty*—is a far more direct measure of material disadvantage than the proportion of residents in a GB LAD identifying as experiencing *not good health*. No equivalent direct measure of poverty is published for the 2011 UK Census at LAD-level. Given this potential conceptual muddling, it is important that in our model specification we also control for the age structure of GB LADs—it is likely that age and *not good health* are closely associated. The share of residents that are *foreign born* and *older adults* (adults aged 65+) are loose measures of ‘age-migrant mix’, variables that particularly characterise the ‘left-behind’ tendency, and (log) *population density* was selected as a variable that separates metropolitan or ‘big city’ contexts. Worth mentioning is the issue that, as with previously published area-level analyses (e.g. [4, 7–9, 14, 18, 19]), these explanatory variables measure outcomes six years prior to the votes. Certain US counties and GB LADs will have experienced greater demographic change over this period than others and, especially for US counties with smaller populations, this may introduce additional uncertainty into our modelling that cannot be easily accounted for.

### Analysis methods

#### Interrogating into regionally-scaled structure

This study uses a regression framework to consider the extent to which area-level variation in populist vote shares can be explained by variation in area-level demographics and structural-economic characteristics. However, we pay particular attention to regional structuring in this variation and allow for the possibility that some contextual variables might explain variation better and differently in different parts of GB and US.

Associations between individual outcome and explanatory variables are explored via standard univariate linear models, updated to recognise a regional hierarchical structure. Here, the *net-Leave*, *net-Trump* and *shift-Trump* vote in each GB LAD or US county is expressed as a linear function of an area-level demographic or contextual variable. The standard model is first extended by adding a random intercept on region (GB GOR or US state): the distribution of explanatory and outcome variables is allowed to vary from region-to-region but their effect is assumed to be constant (remains fixed). This update is sensitive to the fact that a variable’s *range* may be regionally constrained. The model is then further extended to allow the slope as well as intercept to vary on region, enabling analysis of the extent to which associations between variables may change by region explicitly.

#### Controlling for confounders and exposing regionally-specific explanation

In the second section of our analysis we develop full multivariate explanatory models: we investigate the effect of our selected variables after controlling for variation in each. Models are first built globally (without controlling for regional structure), then by modelling the subnational scale as fixed effect dummy variables (as per the existing literature) and finally by developing an ensemble of models built separately for each region (all 11 GORs of GB and 48 states of mainland US and District of Columbia).

Ultimately in regression modelling the challenge is to derive model solutions that are easy to interpret with little redundancy in explanatory variables (parsimony), and that explain the outcome reasonably well and reliably (e.g. bias-variance trade-off [27]). For models estimated using Ordinary Least Squares (OLS), regression coefficients are derived when the difference between modelled predictions and the true outcome (model bias) is minimised. Using OLS estimates of model fit alone to inform model specification and variable selection is problematic. As explanatory variables are added, model fit can increase slightly or substantially, but will never decrease. A common scenario is that a model specified with many explanatory variables fits the data well (bias is low), but contains coefficients that are inflated in magnitude and that likely *vary* between different model realisations. This can happen where several variables are correlated and a large positive coefficient on one variable is negated by a similarly large coefficient in the opposite direction in a related variable [27]. These problems are typically addressed in Social Science applications by judicious variable selection and computing coefficient Variance Inflation Factors (VIF). Such a practice is acceptable in use cases where one or two models are to be generated. However, in this study we wish to generate and compare across many regional models (11 for GB and 49 for US). Problems between variables identified at the US or GB scale may not exist at the regional scale and the reverse may also be true—new problems may be identified at the regional scale that do not exist at the US or GB scale. After *a priori* variable selection, performing further selection via inspecting VIF scores from multiple candidate model specifications that are then built separately for c.100 regional models (models are fit with both *net-Trump* and *shift-Trump* as outcomes) is problematic and it is for this reason that we deviate from a multilevel modelling framework in favour of penalised regression.

Penalised regression is similar to OLS in that it tries to maximise model fit in the same way; however it also adds an upper bound, or penalty, on the sum of regression coefficients, penalising against those likely to be unstable. This penalty can be differently applied. In *ridge regression*, variables containing coefficients with extreme values are shrunk continuously towards zero [28]; in *least absolute shrinkage and selection operator* (LASSO) penalised coefficients can be shrunk to zero and removed from the model entirely [29]; and *elastic-net* [10] uses a combination of the two. The high-level difference between these can be understood when considering their treatment of correlated variables. Presented with a pair or group of highly correlated variables, ridge will shrink the coefficients of all variables in the group; LASSO will pick one or a small number of variables in the group and drop the rest but can be indifferent to the variables that are picked or dropped; elastic-net combines both penalties, shrinking the coefficients in the group and performing variable selection simultaneously. An important point to emphasise given our use case is that penalised regression provides a principled approach to automatic variable selection that can be applied at scale (for the 100+ regional models)—it generates model solutions that are concise with parameters estimates that are more reliable under replication [11].

In order to fit our c.100 regional models we use elastic-net. Since elastic-net imposes constraints on regression coefficients, we *z* − *score* standardise our explanatory variables before running the procedure and calculate measures of uncertainty around the parameter estimates via a bootstrap. Model outputs are presented in Figs 3, 4, 5 and 6. A consistent mapping of regression coefficients is used in each figure, and for the regional model outputs plots are arranged semi-geographically as small multiples (e.g. [30]) in order support spatial comparison.

There are limits to how fully we can document analysis decisions in this paper. Further discussion, with additional analysis and presentation of sensitivity checks around the penalised regression models, is provided in the paper’s accompanying github repository.

## Analysis

### Area- and region- level variation in voting behaviour

**Fig 1:**
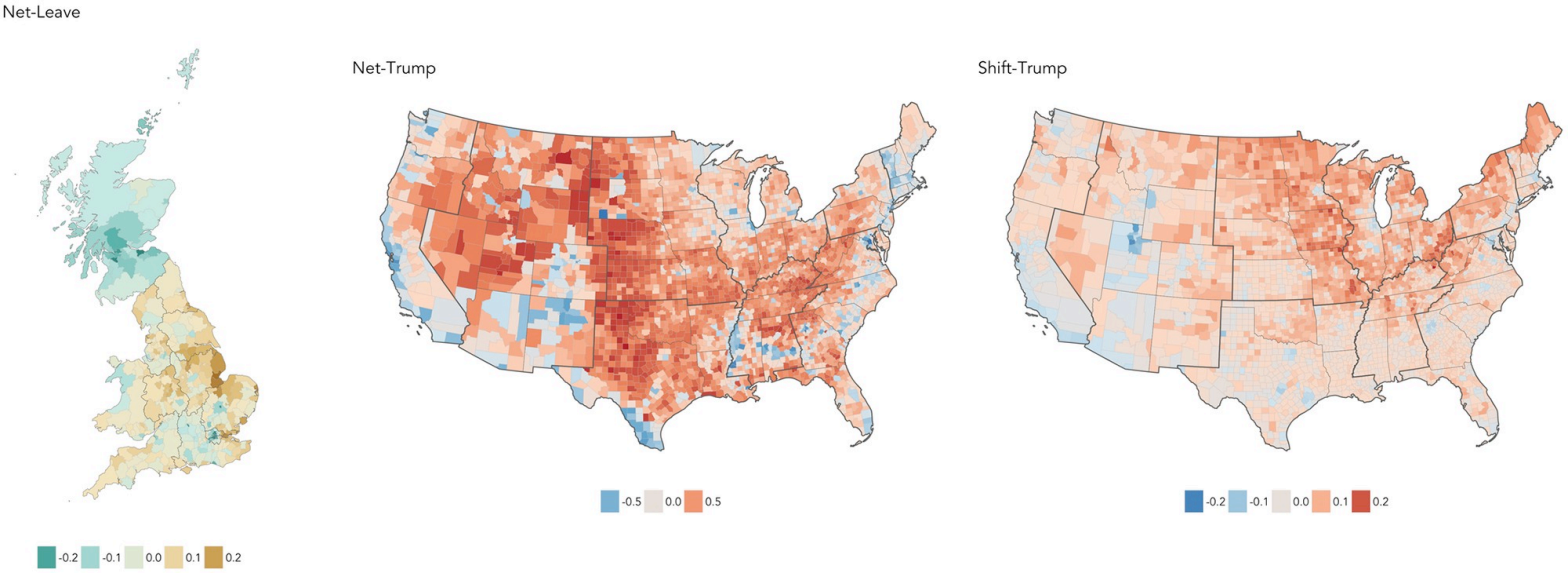
Choropleths of the three outcome variables aggregated to GB LAD and US county level. Left: majority Leave is brown, Remain is green. Middle: majority Trump is red, Clinton is blue. Right: shift towards Trump is red, away is blue.

Fig 1 shows difference maps for the votes: GB LAD net vote shares for Leave|Remain (*net-Leave*), US county two-party net Trump|Clinton vote shares (*net-Trump*) and vote shift from 2012 in favour of Trump (*shift-Trump*). Net direction in the area-level majorities is differentiated using colour hue and the size of margin using colour lightness (darker colours are associated with larger vote margins).

The maps demonstrate that voting behaviour on these outcomes has a very distinct geography. For GB, the Remain vote concentrates heavily in Scotland and London; excluding London, just 18% of LADs in England & Wales voted majority Remain. Whilst the middle map is a reasonably familiar pattern of Republican versus Democrat voting, there are departures from a ‘typical’ patterning to a US election. These are emphasised in the right map. Although *shift-Trump* was present across much of the US (87% of counties experienced an enlarged Republican vote share from 2012), the extent of shift is most apparent in historically blue-collar counties located around the Great Lakes area and rural North East. Also notice counties in California, which despite voting Republican moved away from Trump. Less easy to identify is the further shift from Trump, which became even more emphatically Democrat, in generally larger metropolitan counties.

### Regionally-scaled associations between area-level context and voting

Given the obvious area-level and regional patterns in voting behaviour, we can justifiably ask whether this variation in voting preference also varies with demographics and local contexts in consistent ways. We start by exploring associations between our outcome variables, *net-Leave*, *net-Trump* and *shift-Trump*, and candidate explanatory variables aggregated to county and LAD-level.

**Fig 2:**
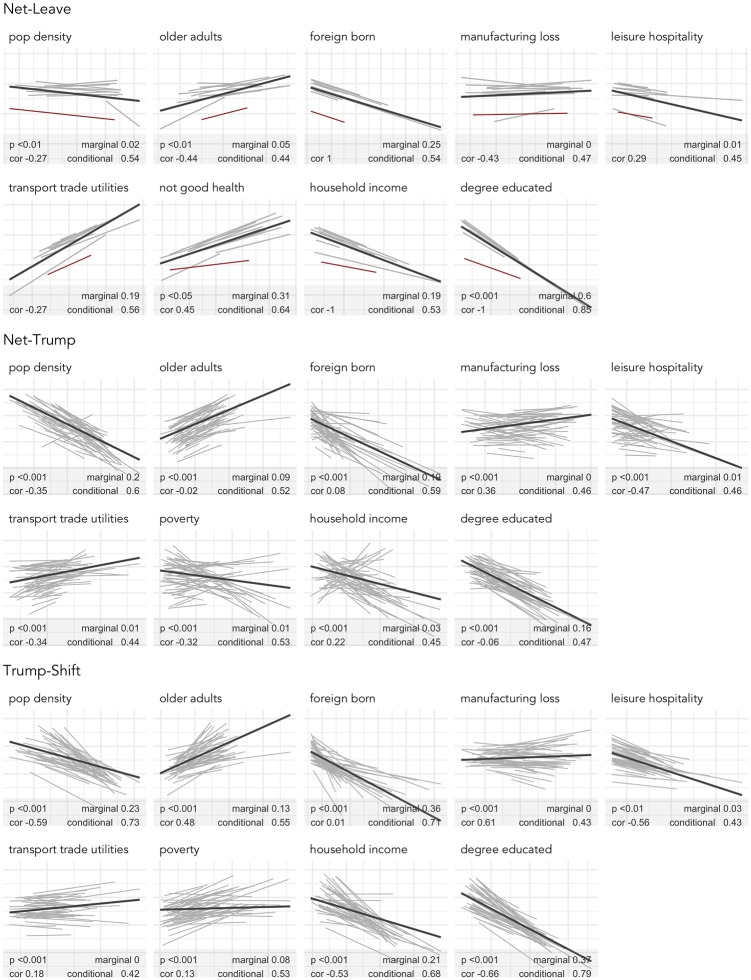
Multilevel models fit to each explanatory variable for *net-Leave*, *net-Trump* and *shift-Trump*. The overall regression line is bold and regional slopes grey (Scotland is identified with a red line). Plots are annotated with estimates of pseudo-*R*^2^, *p-values* from likelihood ratio tests comparing varying slope with varying intercept models and correlation values between slopes and intercepts for the random slope models.

Fig 2 shows univariate multilevel models fit separately to each explanatory variable assumed to be discriminating. The bold lines in the figure are the overall regression line and in grey are separate lines where we allow the slope and intercept to vary on GOR and state. Parallel slopes with little variation in vertical position imply that associations are not regionally structured; parallel slopes with large variation in vertical position suggest that associations *are* regionally structured (or scaled) but a consistent association between variables exists; substantial changes in slope (cluttered display) suggests both regionally-specific scale and pattern of associations. Also labelled on each plot are statistics for evaluating the nature of regional variation. The *p-value* is derived from a likelihood ratio testing the random slope against random intercept models (although not presented in the figure, models were also fit with only a random intercept term). If a difference exists between the random intercept and slope models, there is further evidence to suggest that associations between variables, and not just variable ranges, are subject to change on region (e.g. slopes differ by region). Two measures of pseudo-*R*^2^ are presented [31]. Marginal *R*^2^ describes the proportion of variation explained by fixed effects alone; conditional *R*^2^ the proportion of variation explained by both fixed and random effects. Large differences between marginal and conditional *R*^2^ therefore suggest strong regional structuring.

The most discriminating covariate (*degree-educated*) measures the proportion in a GB LAD or US county educated at least to Bachelors level. As this quantity increases, *net-Leave*, *net-Trump* and *shift-Trump* decreases. For GB this is essentially a ‘global’ covariate—excluding Scotland (highlighted), there is comparatively little regional structuring. For the US, after accounting for regional differences in the range of this variable, there is a consistent direction of association with *degree-educated* and the *net-Trump* and *shift-Trump* outcomes but notice that, as with Brexit, the association is more emphatic for *shift-Trump*. Associations in the same direction can be identified for *household income*, *leisure and hospitality* and share of the population that are *foreign born* and *population density*, consistent with the ‘left-behind’ interpretation. The fact that the association with *household income* is both more consistent and large with *shift-Trump* as the outcome, and not with *net-Trump*, which is more heavily conflated with standard Republican voting, reinforces the link between nascent voting shifts in favour of a ‘populist’ agenda and disadvantage. Positively associated with the outcome variables are *transport, trade & utilities*, *older adults* and *manufacturing-loss* (though not for *net-Leave*). An important point of departure between *net-Trump* and the outcome variables assumed to be more direct measures of ‘populist’ voting (*net-Leave* and *shift-Trump*) is on the material disadvantage variables. Whilst there is a relatively strong positive association with *not good health* and *net-Leave*, a positive association (though subject to regional change) with *poverty* and *shift-Trump*, there is in fact an overall negative association with *poverty* and *net-Trump*.

The regional specificity in associations, implied by the multilevel slopes and confirmed by conditional and marginal pseudo-*R*^2^ in Fig 2, further justifies the approach taken in this study. Estimating separate slope and intercept parameters on state and GOR substantially reduces the residual variation in the univariate models. For GB, all variables exhibit regional *scale*—although Scotland (highlighted) contributes disproportionately to the differences implied by the marginal and conditional *R*^2^. According to the likelihood ratio test comparing random slopes with random intercepts, there is evidence of differing *associations* with the outcome (*net-Leave*) for *population density*, *older adults* and *not good health*. Regional differences in both the *scale* and *assocation* between variables are more evident in the US. This is especially true of *poverty*, *household income* and *manufacturing loss*. Notice that overall, *poverty* is negatively associated with *net-Trump*—as the relative proportion of residents living in *poverty* increases, counties’ *net-Trump* vote decreases—but that there are several states for which there is a positive association. Importantly, this tendency towards a positive association for *poverty* amongst particular states is more evident where *shift-Trump* is the outcome. Notice also that of the industrial structure variables, *manufacturing loss* is the variable most subject to regional change in its association with *net-Trump* and *shift-Trump*.

### Global associations between area-level context and voting after controlling for confounders

**Fig 3:**
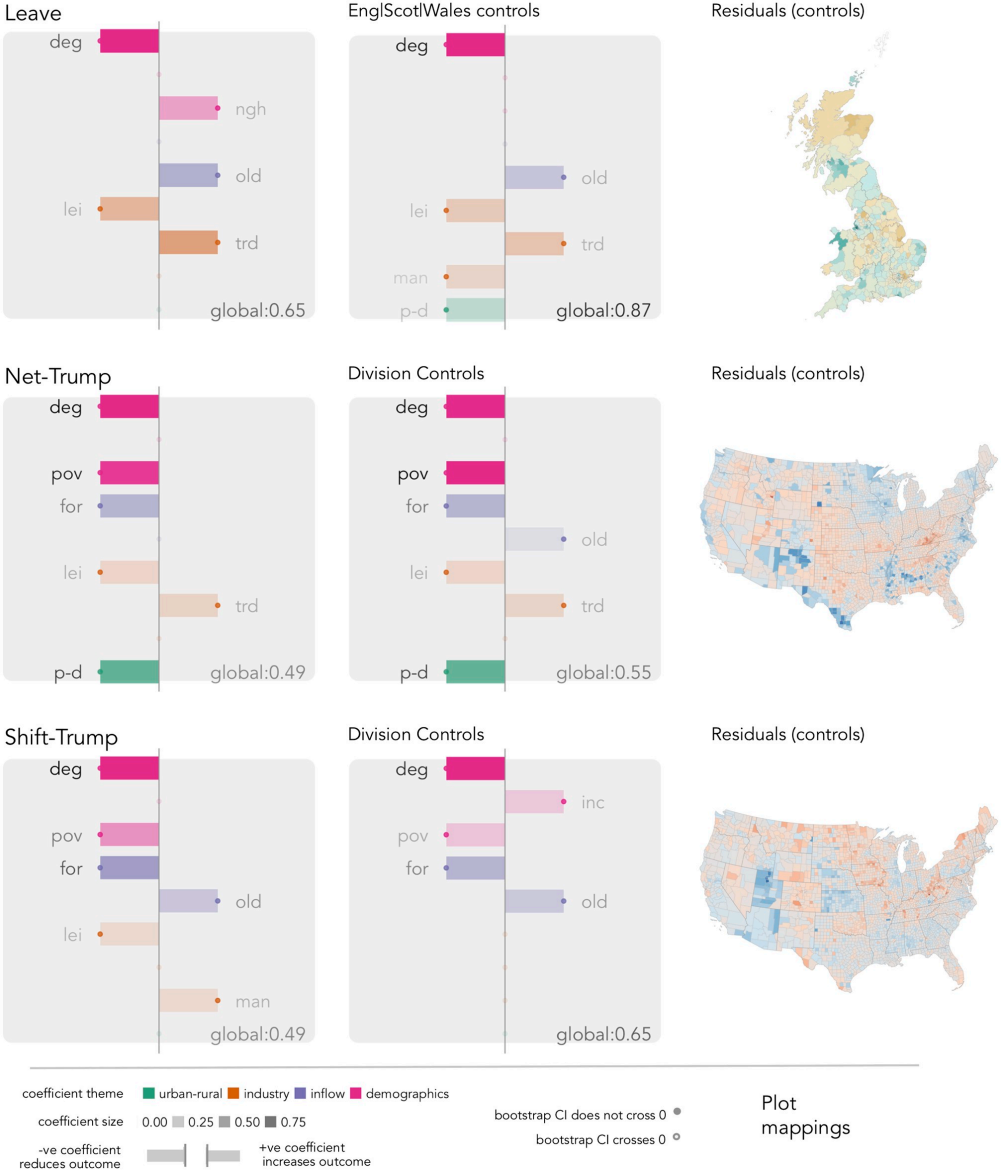
Coefficients for multivariate models fit using elastic-net. Each variable selected is labelled and identified with a bar; bars left of the vertical represent negative coefficients and right of the vertical positive coefficients; bar and variable label lightness varies according to coefficient size; and if the 95% bootstrap confidence interval around the coefficient does not cross zero (e.g. the coefficient is statistically significant) is accompanied with a filled dot. In the bottom right of each plot are estimates of adjusted *R*^2^. Left: summary of global models fit without subnational controls; middle: summary of global models fit with subnational controls (England, Scotland and Wales for GB, the nine Census divisions for US); right: choropleth of residuals for models fit with subnational controls. Green—Leave vote is lower than expected; Blue—*net-Trump* and *shift-Trump* is lower than expected.

Presented in Fig 3 are coefficients from multivariate models specified with *net-Leave*, *net-Trump* and *shift-Trump* as outcomes and fit using elastic-net. In the first column all nine variables are passed to the elastic-net procedure, but the model is not specified to account for regional structure. In the second column, subnational controls are added as fixed effects as in Essletzbichler et al. [4]. LADs are grouped according to the three nations of GB—England, Wales and Scotland—and US counties are grouped according to the nine Census divisions of the US—New England, Mid-Atlantic, East-North Central, West-North Central, South Atlantic, East-South Central, West-South Central, Mountain and Pacific. We consider any change to estimated coefficients in our nine explanatory variables as well as improvements in model fit. In the right-most column are maps of residuals for the models specified with subnational controls.

The GB model broadly aligns with expectations. *Degree educated* is the largest covariate: after controlling for variation in *older adults*, *leisure & hospitality*, *transport, trade & utilities* and *not good health*, an increase in the proportion of residents in a LAD educated at least to degree-level has the effect of reducing the *net-Leave* vote. The reverse is true of *older adults* and *not good health*, variables we assume represent less mobile and prosperous populations, and dependence on *transport, trade & utilities*, a sector particularly at risk to automation. The addition of subnational controls results in an improved model fit and a broadly similar pattern of associations with the outcome—although *not good health* becomes less important and is excluded, *population density* and *manufacturing loss* are added with small negative coefficients. Strikingly, even after including the subnational controls there is strong spatial autocorrelation in model residuals: particularly for parts of the North West (Liverpool, Sefton, Manchester, Wirral) the Leave vote is lower than would be expected given its demographic and economic structure.

There are similarities between the GB model and coefficients identified in the *shift-Trump* and to a lesser extent *net-Trump* model; a related set of variables are selected and in each *degree-educated* remains the most important covariate. There are nevertheless exceptions. Added to the *net-Trump* model is *population density* and *foreign born*; and to *shift-Trump* is *manufacturing loss*. The direction of effect in these variables is important. After controlling for levels of *degree* education and variables such as *population density* that distinguish metropolitan from peripheral counties, *poverty* serves to reduce the votes—and with a large negative coefficient—for *net-Trump*. During variable collection, *not good health* was used in GB as a loose substitute for *poverty*. We assumed parity between the two—that both represent material disadvantage. That a difference between GB and US is observed in these variables, after controlling for key confounders (levels of education, industrial structure and *older adults*), we may infer that populist voting is less a function of area-level material disadvantage in the US than it is in GB. An alternative reading is that the *Leave* vote is more obviously separate from the legacies of political party affiliations than is *net-Trump* and *shift-Trump*, which essentially represents an endorsement of a Republican candidate. This is partially supported by the fact the coefficient for *poverty* in the *shift-Trump* model is much smaller than that estimated for *net-Trump*. Certainly exploring how this variable is reported in state-level models would be instructive. Notice again that even with the inclusion of subnational controls, residuals are spatially autocorrelated (choropleths in right column of Fig 3).

### Exposing regionally-specific explanation

**Fig 4:**
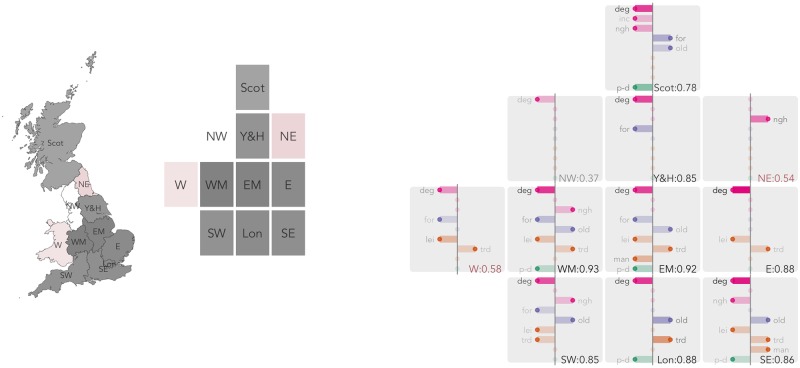
Semi-spatially arranged small multiples of outputs for models fit separately to each GB GOR. The plot mappings described in Fig 3 are repeated. To the left, a choropleth and spatially arranged small multiple clarifies the geographic ordering to GORs and filled according to model fit (the darker the fill, the greater the model fit). For a GOR containing fewer than 30 LADs, too few for parametric assumptions to hold, we add observations from neighbouring LADs (based on centroid-to-centroid distances) until that GOR contains 30 observations. These special cases are identified with red text labels and fill.

In this section, we present models fit separately for each GB GOR and mainland US state, again using elastic-net for variable regularisation and selection. This procedure results in a detailed set of model outputs—separate coefficient estimates for unique combinations of variables across 11 GB GORs and 48 US states (plus District of Columbia). Not only do we wish to explore whether suggested model solutions vary between GORs and US states but also whether there is spatial dependency to this variation—whether particular categories of model solution (combinations of variable) are suggested for different parts of the country. We repeat the model summary plots used in Fig 3, but arrange each in their approximate geographic position (see Meulemans et al. [30] for a discussion of such approaches). These are presented in Figs 4 (*net-Leave*), 5 (*net-Trump*) and 6 (*shift-Trump*). Note that we do not discuss in detail the models with *net-Trump* as the outcome. This is partly for reasons of space, but also the fact that, as demonstrated in the analysis above, *shift-Trump* more closely aligns with *net-Leave*.

**Fig 5:**
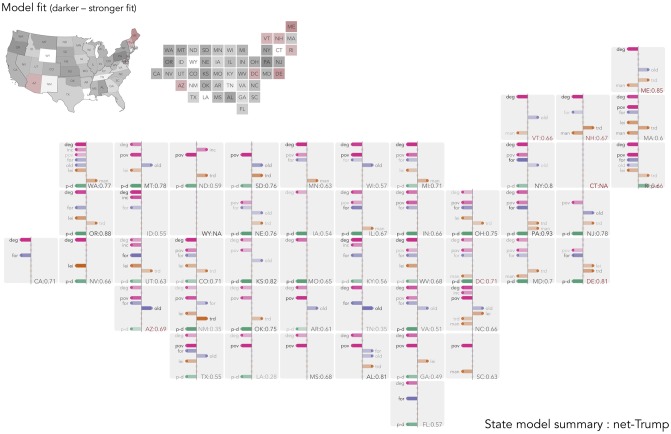
Semi-spatially arranged small multiples of outputs for models fit separately to each US state. The plot mappings described in Fig 4 are repeated. For US states containing fewer than 30 counties, we add observations from neighbouring counties (based on centroid-to-centroid distances) until that state contains 30 observations—identified with red text and fill.

**Fig 6:**
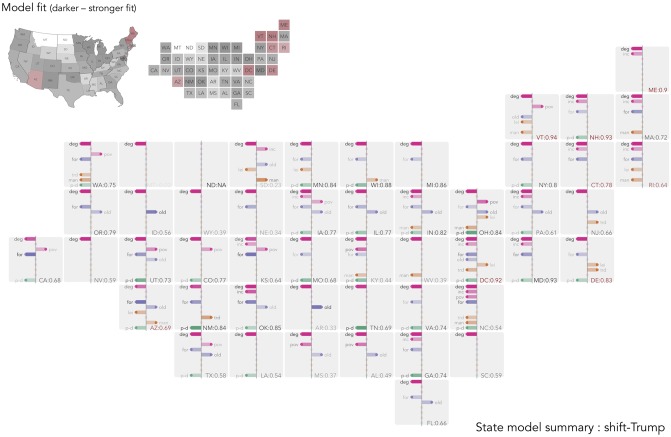
Semi-spatially arranged small multiples of outputs for models fit separately to each US state. The plot mappings described in Fig 4 are repeated. For US states containing fewer than 30 counties, we add observations from neighbouring counties (based on centroid-to-centroid distances) until that state contains 30 observations—identified with red text and fill.

#### Regionally-specific explanation in GB

Several observations can be made from the regional models of the Brexit vote. Again *degree-educated* remains the dominant covariate. With the exception of the North East region, *degree-educated* is consistently selected by the elastic-net procedure with a large coefficient and its effect is in the expected direction. Variables that describe the industrial structure of regions (orange/brown bars) also align with expectations: *transport, trade & utilities*, a variable that represents dependency on industries vulnerable to automation, and *manufacturing loss* serve to increase the *net-Leave* vote and *leisure & hospitality* reduce the *net-Leave* vote. Notice that no industrial structure variables are selected for Scotland, North West, Yorkshire & Humber.

In England & Wales, variables related to migration and age mix (*older adults* and *foreign born*) are selected with coefficients in the anticipated direction—*older adults* increases the *net-Leave* vote whereas *foreign-born* reduces the *net-Leave* vote. On these variables, a slightly different pattern of associations is suggested for Scotland. After controlling for variation in other covariates, *foreign-born* in fact reinforces the *net-Leave* vote. Also, *not good health* and *household income* are selected and assigned a negative coefficient. Given this pattern is unique to Scotland, this might suggest that area-level disadvantage informs political preference differently in Scotland to other regions of GB. Clearly, political attitudes towards EU membership in Scotland diverge greatly with the rest of England and Wales—and this has been exposed in ecological studies through analyses of model residuals (e.g. [18]). That this pattern of associations is unique to Scotland, we can add that area-level disadvantage variables appear to inform area-level Leave voting differently in Scotland and, as a corollary, that the ‘left-behind’ reading may apply less strongly here.

Also interesting is the fact that for several regions (Yorkshire & Humber, North East, West Midlands, East Midlands and South West) *not good health* is selected and with a positive coefficient: after controlling for industrial structure, levels of education and demographic mix (*older adults* and *foreign born*), *not good health*, the variable we use as a proxy for material disadvantage, serves to increase the *net-Leave* vote. The largest coefficient for *not good health* is in the North East region, selected as *the* most important covariate and it should be noted that with the exception of the South West, these are regions of England that experienced the greatest loss in the relative size of manufacturing employment between 1971 and 2011. An additional observation here is that in our sensitivity analysis (detailed in the github repository), *household income* appears with positive coefficients and *not good health* with negative coefficients. This observation should be treated cautiously since the coefficients are small and contain uncertainty. Nevertheless, a pattern of associations for the South East where material disadvantage instead reinforces the Remain vote is interesting; counties in the South East region (the ‘home counties’) have previously been identified as containing political traits and historical associations with euroscepticism characteristically distinct from other parts of the country, particularly the north and midlands [32].

There are differences in the success with which the regional models explain variation in the vote. Whilst *net-Leave* did vary between the 39 LADs located in the North West (Manchester was 60% Remain, Blackpool 67% Leave), only 37% of this variation can be explained by the local context variables. There is also a reasonably large amount of residual variation for models fit to the Wales and North East regions. Finally, the model for the East of England is unique (with the exception of the North East) in that no variables related to migration and age mix (*older adults* and *foreign born*) are selected. A very large share of the variation in the vote (90%) can be explained by *degree-education* and *industrial structure* variables. Although the East region contains some of the most pro-Leave LADs in GB, especially so towards the coast (Castle Point 73% and Thurrock 72% for Leave), it contains some strong pro-Remain areas with highly contrasting demographic contexts and economic histories (Cambridge 74% and St. Albans 63% for Remain).

#### Regionally-specific explanation in US

For the state-level models built on *shift-Trump*, *degree-educated* is again *the* covariate, with the largest coefficient value and a consistent direction of effect in each of the state-level models. In this, the state-level models on *shift-Trump* mirror those of the Brexit vote. A consistent pattern also exists for *foreign-born* (appears in 32 of the 48 state-level models) and *population density* (in 27 of the models) with the direction of effect aligned with expectations: after controlling for variation in other context variables, more densely populated counties are associated with a reduced *shift-Trump* vote; so too for counties associated with comparatively large *foreign-born* populations. *Older adults* is also selected in 19 of the models and, with the exception of Vermont, in the anticipated direction.

*Poverty* is the variable most subject to regional change. This variable appears in 13 of the state-level models but in only four of these does it follow the global model and have the effect of reducing the *shift-Trump* vote. There is some geography to this varying effect, especially when interpreted alongside the *household income* variable. A negative coefficient is assigned to several southern states (Mississippi, Alabama, North Carolina, Kentucky) and this pattern is replicated more strongly in the state-level models built with *net-Trump* as the outcome (notice in these models that *poverty* is of equal saliency to *degree-educated*). For several other states in the country, but particularly the West Coast (California and Washington), New England and the Mid Atlantic, however, either this variable serves to increase the *shift-Trump* vote, or the *household income* variable, which appears in 15 models, is selected and given a negative coefficient, reducing the *shift-Trump* vote. This pattern of associations was hidden by our global model which suggested, as per models derived for southern states, that *poverty* serves to reduce the *shift-Trump* vote. The fact that the direction of effect appears to change in a geographically systematic way is further evidence of change in process—of regionally specialised effect.

Variables describing industrial structure tend to appear less often and again in particular parts of the country and different to expectation given the global model. *Manufacturing loss* is the most frequently occurring of these variables, appearing in 13 state-level models. Its effect on the outcome, however, aligns with expectation in only three of these: after controlling for demographic structure, education levels and *population density*, *manufacturing loss* has the effect of increasing the *shift-Trump* vote for South Dakota, Wisconsin and Arizona. Elsewhere, this variable appears with a negative coefficient. Interestingly, the industrial structure variables appear far more frequently and in line with expectation in the local models built on the *net-Trump* outcome. For states in the Great Lakes region (Michigan, Minnesota and Illinois) *manufacturing loss* is selected in the *net-Trump* models and with a positive coefficient and elsewhere, *transport, trade & utilities*, the variable we associate with industries vulnerable to automation, appears on 19 occasions and overwhelmingly (in 18 of these) with a positive coefficient. There is some evidence of similar patterns of association with *shift-Trump* as the outcome where for Wisconsin, South Dakota and also Arizona *manufacturing loss* is selected with positive coefficients; however in nine states this variable is assigned a negative coefficient. For *shift-Trump*, then, socio-economic characteristics and population dynamics are more stable and important explanatory variables than those characterising industrial structure and history.

## Discussion

### Empirical contributions

This study mostly reinforces existing ecological analyses of recent populist voting and the widely cited narrative of ‘left-behind’ places. Studying the Brexit vote in UK and Trump vote in US, we find associations and model outputs that would be expected given this interpretation. By far the most important variable is that measuring levels of *degree* education: net of other demographic and structural-economic characteristics, as the share of residents in an area educated at least to degree-level increases, the populist vote share decreases. We can add that this is a ‘global’ variable to the extent that it contributes the largest effect and does so consistently across regions of GB and US. That this variable is comparatively less important for models built using *net-Trump* as an outcome, a measure more heavily conflated with traditional Republican affiliations, further confirms *degree-educated*, and the concepts and behaviours it represents, as discriminating the new ‘populist’ tendency.

Despite this variable’s dominance, secondary variables do contribute an effect—but these effects are smaller and more prone to regional variation. Variables describing migration characteristics and separating big city contexts tend to work in predictable ways; *foreign born* and *population density* reduce the populist vote and *older adults* increase it, although we find some interesting exceptions. Those variables describing area-level dependency on jobs vulnerable to automation and experiencing *manufacturing loss* also contribute towards the models, but appear less frequently and with smaller and less geographically consistent effects than might be expected given the ‘left-behind’ reading. Variables relating to material disadvantage have the most interesting pattern of effects. For the US, area-level *poverty* and (lack of) *household income* reduces the *net-Trump* vote, particularly so for southern states. This pattern of association is present in global models built on *shift-Trump*, an outcome that more closely approximates to the populist element, and partly undermines the ‘left-behind’ explanation. However, our region-level models uniquely reveal not only that the size of these effects vary, but that the direction effects are subject to change. For southern states of US the negative effect persists, but in many northern states, particularly those in New England and Mid-Atlantic, variables representing material disadvantage assist the Trump vote. There is also a geography to these variables in regional models fit to the Brexit vote: material disadvantage variables are selected and with positive coefficients (increasing the *net-Leave* vote) in regions whose employment previously depended on traditional industries—and it is here where our regional model outputs suggest parallels between the US and GB case.

### Methodological consequences

Implications can be drawn from these findings, and particularly our research design, that are prescient to ecological studies of populist voting behaviour, but also Social and Political Science analysis more generally.

First, since levels of (degree) education are so discriminating and robust to geography, then analyses that prioritise some uniquely-defined variable combination in contributing new explanations behind the votes should be reported cautiously. In addition to the usual concerns around ecological fallacy and inflated model fits [33], in an area-level analysis selected variables are only loose approximations of wider concepts. The ‘*pet-variable problem*’, where a researcher focuses on a favoured explanatory variable and *p-hacks* [34] their way to demonstrating its statistical significance from a carefully constructed model specification [11] is to be avoided. Our application of penalised regression and use of computational techniques for deriving uncertainty estimates (bootstrapping) helps guard against this by eschewing complex model specifications and coefficients that are unlikely to replicate (see [11]).

Second is the treatment of geography. The results of our regional exploration are in many ways reassuring; we did not discover a radically different pattern of associations and model solutions to those already presented. The fact that we were able to identify deviations that were spatially systematic nevertheless allowed some new insights into the phenomena: for example, those around the material disadvantage variables. Rather than ‘control[ing] away’ [12, 17] unknown subnational context, we therefore advocate for the use of modelling frameworks that actively support exploration into that context by allowing for spatially-varying effects. Indeed, modelling approaches not constrained by regional administrative geographies, for example geographically-weighted penalised regression [35], may be an instructive future exploratory activity.

Third, and a related point: performing such exploratory analysis is challenging. Even in limiting ourselves to a reasonably high-level administrative hierarchy (US states and GB GORs), it was necessary to reason about the outputs of c.100 models concurrently, whilst also looking for spatial relations. Interrogating over outputs in the *de facto* way, via detailed tables of parameter estimates, was clearly unworkable and our approach of using carefully designed visual mappings, with effect size and uncertainty information intrinsically encoded, could be applied more widely when inspecting regression model outputs.

Finally, that we make efforts towards enabling reproducibility of our work, publishing a github repository of our analysis with detailed sensitivity tests, we hope responds directly to recent critiques around Social and Political Science methodology [11–13, 36], demonstrating transparent research design and supporting cumulative knowledge development.
